# Determining factors of physical activity and sedentary behaviour in university students during the COVID-19 pandemic: A longitudinal study

**DOI:** 10.1371/journal.pone.0298134

**Published:** 2024-02-23

**Authors:** Matthew J. Savage, Daniele Magistro, Philip J. Hennis, James Donaldson, Laura C. Healy, Kirsty A. Hunter, Ruth M. James

**Affiliations:** SHAPE Research Group, School of Science and Technology, Nottingham Trent University, Nottingham, United Kingdom; George Mason University, UNITED STATES

## Abstract

**Introduction:**

Historically, university students demonstrate poor movement behaviours that could negatively impact current and future health. Recent literature has focused on identifying determinants of physical activity (PA) and sedentary behaviour (SB) in this population to inform the development of intervention strategies. However, the COVID-19 pandemic substantially restricted movement behaviours in this population, meaning findings of previous research may no longer be applicable within the current societal context. The present study explored the longitudinal relationships between pre-pandemic psychological, behavioural and anthropometric factors, and the movement behaviours of UK university students nine months following the outbreak of COVID-19.

**Methods:**

Mental wellbeing (MWB), perceived stress (PS), body mass index (BMI), SB, and PA were assessed using an online self-report survey in 255 students prior to (October 2019) and nine months following (October 2020) the first confirmed case of COVID-19 in the UK. Path analysis was utilised to test relationships between pre-COVID mental wellbeing, perceived stress and BMI, and movement behaviours during the pandemic.

**Results:**

The fit of the path analysis model was good (*χ*^*2*^ = 0.01; *CMIN* = 0.10, *CFI* = 1.00, *RMSEA* = 0.00). Pre-covid MWB and PS positively influenced PA (*β* = 0.29; *β* = 0.24; *P* < 0.01) but not SB (*β* = -0.10; *β* = 0.00; *P* = 0.79) during the pandemic. Additionally, pre-pandemic SB and PA positively influenced SB and PA during the pandemic respectively (SB: *β* = 0.26; *P* < 0.01) (PA: *β* = 0.55; *P* < 0.01). Pre-pandemic BMI did not influence any measured variable during the pandemic (PA: *β* = 0.03 and *P* = 0.29; SB: *β* = 0.06 and *P* = 0.56), and there was no mediating effect of PA on SB during the pandemic (*β* = -0.26; *P* = 0.14).

**Conclusion:**

These findings indicate that pre-covid mental health and movement behaviours had a direct positive influence on PA during the pandemic, but not SB. This longitudinal study demonstrates the influence that prior psychological and behavioural factors have in determining university students’ response to periods of elevated stress and uncertainty, furthering our understanding of determinants of health-related behaviours in students.

## Introduction

The health burden of inadequate physical activity (PA) and excessive sedentary behaviour (SB) has been well documented [1, 2]. In 2016, the UK spent almost £1 billion on diseases directly related to low levels of PA and excessive SB, and there were 69,276 avoidable deaths from non-communicable diseases (NCDs) that developed as a direct consequence of chronic SB (≥ 6 hours per day) and insufficient PA [2]. Whilst the repercussions may not be prevalent for years to come, young people may be increasing their risk of developing NCDs given that habitual physical inactivity and prolonged SB begins during early adulthood and is often sustained into mid-life [3]. This is of particular concern in university students who spend an average of 7.3 to 9.8 hours per day sedentary and experience substantially higher weight gain compared to age matched individuals in the general population [4–6]. There is a clear need to improve movement behaviours in university students, but to achieve this, researchers must first understand the determinants of PA and SB in this population.

Whilst related, PA and SB are separate behaviours with independent definitions and consequences. Indeed, previous research has shown that individuals who engage in prolonged periods of SB have a higher risk of developing poorer health outcomes (e.g., reduced lean body mass and upper body strength) and premature death, even when taking part in adequate amounts of PA [7, 8]. Since PA and SB can be differentiated by both their technical definitions and, more importantly, their influence on health outcomes, it is also likely that these behaviours are influenced independently and by a variety of disparate factors. Indeed, several studies have demonstrated that specific factors that are associated with PA are not associated with SB, and vice versa, in young people [9–11]. However, it is unclear whether similar relationships are present in university students given the unique context within which student-hood resides [12–15]. Nonetheless, a previous ecological framework has outlined that intra- and inter-personal factors all have an influence on university student’s PA and SB [16], although, only intra-personal non-modifiable factors (e.g., gender) have been investigated sufficiently to identify correlations in this population [17]. Specifically, women have been shown to spend more time sitting and engaging in screen time than men [17] and men are more likely to take part in organised bouts of PA than women [18]. Additionally, there is some evidence to suggest that psychological, behavioural and anthropometric factors are longitudinally related to PA in university students [19–22] and SB in young people [23–25]. However, as concluded by Castro et al., (2018) [17] and Thorley (2017) [26], there remains a lack of multifactorial longitudinal studies in the area and therefore the determinants of SB and PA in university students are yet to be established.

To add further complexity to this issue, the COVID-19 pandemic led to governments imposing restrictions on movement and social contact in their populations. University campuses closed and students were forced to make rapid changes to their living arrangements [27]. They moved to remote learning [28], experienced financial hardship through job loss [29], developed fear of infection and transmission [30], and had reduced capacity to engage in social interaction or obtain emotional support [31]. Consequently, in university students from the UK, substantial increases in SB (+42%) and decreases in PA levels (-11%) were observed during the early stages of the pandemic and were sustained nine months later [32, 33]. Furthermore, the pandemic negatively impacted student’s mental health [32–34] and a substantial proportion of students’ (20–30%) experienced weight gain [35]. Therefore, previous studies exploring factors influencing movement behaviours in young people may no longer be applicable following the COVID-19 pandemic.

Crucially, the context in which the pandemic resides continues to shift; there have not been any COVID related restrictions in the UK for over a year and the general population are now transitioning into a post-COVID society. Yet, despite the lifting of ‘lockdown’ restrictions, it appears that movement behaviours remain impaired in comparison to pre-lockdown levels among both children and adults [36]. Whilst the reasons for this are currently unclear, it may be that there are contextual similarities during and after the COVID-19 pandemic that are negatively influencing movement behaviours such as hybrid working, fear of infection & transmission, and overwhelmed support services [37]. Therefore, identifying pre-COVID factors that influenced movement behaviours during the pandemic could provide critical insight into factors affecting sedentarism and PA post-pandemic. In turn, this could aid in developing strategies to re-establish and improve levels of PA and SB.

Overall, university students demonstrate poor movement behaviours, and this issue has been exacerbated further by the COVID-19 pandemic [32, 33]. Students are now at greater risk of developing poor health-related habits that can negatively influence outcomes of current and future health [7, 8]. To begin to address this problem, it is vital that researchers understand the determinants of movement behaviours within the context of the pandemic. The aim of the current study was therefore to explore, using path analysis derived from structural equation modelling (SEM), the longitudinal relationships between mental wellbeing (MWB), perceived stress (PS), and BMI prior to the pandemic, and SB and PA nine months following the initial outbreak of COVID-19 in UK university students. It was hypothesised that better mental wellbeing, lower levels of perceived stress, and lower pre-pandemic BMI would predict higher amounts of PA and lower levels of SB during the pandemic.

## Materials and methods

### Study participants

Participants were university students from an institution in the East Midlands, UK that were enrolled in a longitudinal cohort study aiming to explore the health and well-being of university students. 255 university students completed an initial survey in October 2019 (T1) and a follow-up survey in October 2020 (T2). Exclusion criteria included any person under 18 years of age and not a student enrolled at Nottingham Trent University. All subjects were informed of the experimental procedure and provided written informed consent prior to participating in the investigation. Data were pseudonymised and remained confidential throughout the study. Ethical approval was granted by the School of Science and Technology Non-Invasive Ethics Committee of the university (application ID: 19/20-76). The study was conducted in accordance with the principles expressed in the Declaration of Helsinki and using STROBE guidelines [38].

### Outcomes

The survey included questions to obtain self-selected sociodemographic information and a question surrounding diagnosed long-term health conditions, as well as the Exercise Vital Sign (EVS) questionnaire to determine levels of moderate to vigorous physical activity (MVPA) [39], and a previously validated question to quantify levels of SB [40]. The survey also included two scales, Cohen’s Perceived Stress Scale (PSS) [41], and the Warwick-Edinburgh Mental Wellbeing Scale (WEMWBS). The PSS uses a 5-point Likert scale (0 = ‘Never’ to 4 = ‘Very often’) with outcome scores ranging from 0 to 40 where higher scores denote greater levels of perceived stress. The WEMWBS also uses a 5-point Likert scale (1 = ‘None of the time’ to 5 = ‘All of the time’) with outcome scores ranging from 14 to 70 where higher scores denote greater mental wellbeing. Both scales were chosen because they have been validated in UK university students and show good reliability and reproducibility (Cronbach’s alpha = 0.89 & Composite reliability (*ρc)* = 0.88) [42, 43].

### Statistical analysis

Normal distribution was assessed via skewness and kurtosis, all variables indicated skewness values between −1 and +1 and kurtosis values between -2 and +2 and were therefore considered normal [44]. Linearity and homoscedasticity were assessed through scatterplots and residual plots, and P-P plots were used to check normality for residuals.

**Fig 1:**
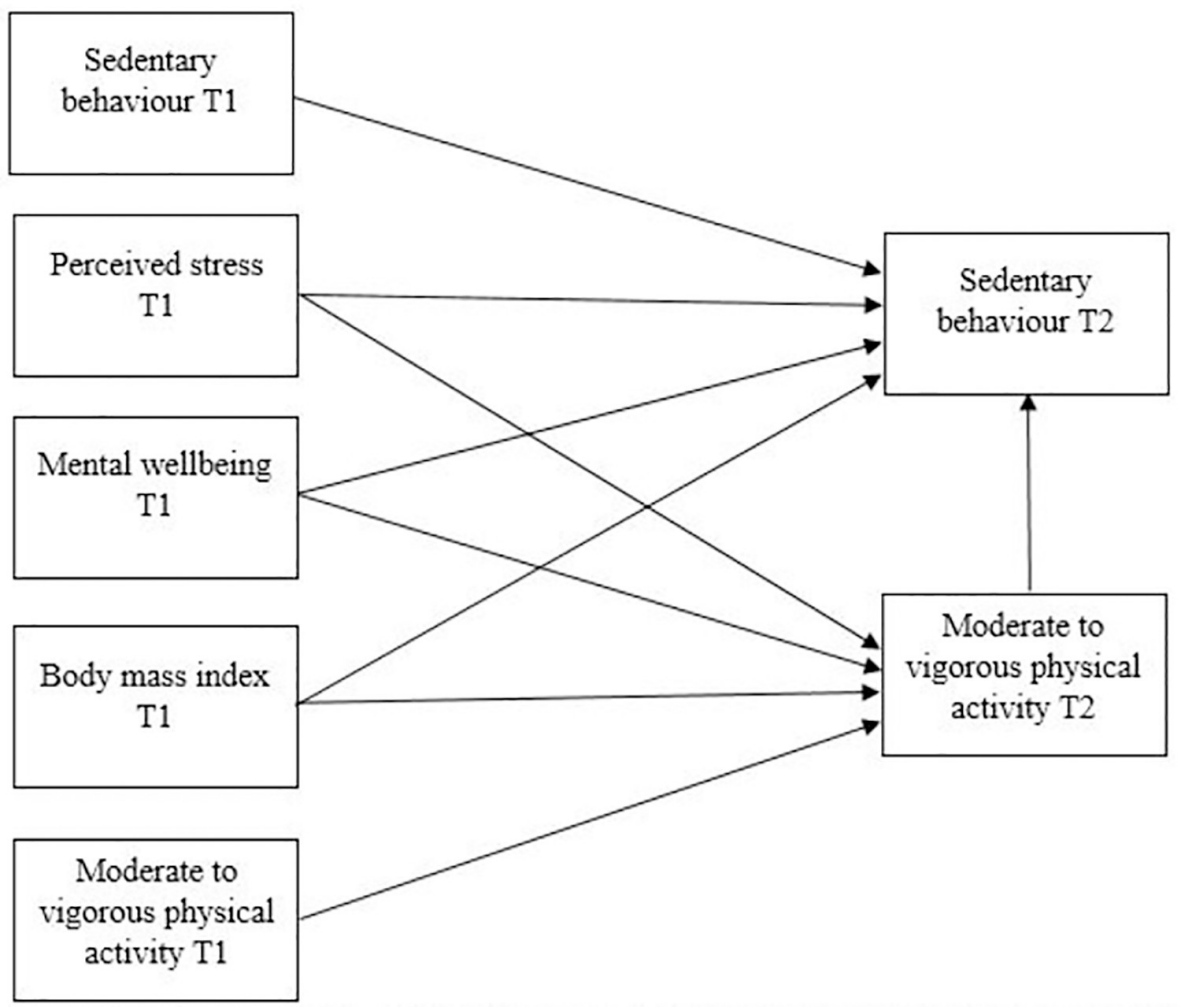
A proposed theoretical model of the longitudinal interactions between mental wellbeing, perceived stress, body mass index, sedentary behaviour, and moderate to vigorous physical activity pre-covid (T1), and sedentary behaviour and moderate to vigorous physical activity during covid (T2).

Multiple group path analysis was conducted using AMOS (AMOS^TM^ version 7.0, Amos Development Corporation, Crawfordville, FL.) to assess the interaction between variables and then to explore the influence of gender using unconstrained estimation. Unconstrained estimation is a straightforward approach that highlights the causes of inadequate fit. It evaluates the model-implied covariance structure, and the unconstrained chi-square test confirms the compatibility of the suggested covariance structure with the observed data [45]. This facilitated the comparison of regression weights between men and women. A proposed model of pre-COVID factors influencing SB and PA during the pandemic is outlined in **Fig 1.** The fit of the model was determined by multiple fit indices; χ^2^ (CMIN), discrepancy divided by degree of freedom (CMIN/DF) where the DF for the general model was 1 and the DF for the model separated by gender was 2 [46], comparative fit index (CFI) and root-mean-squared error (RMSEA). The model was also then separated by gender. Significance was accepted when *P* < 0.05. Squared multiple correlations (*R*^*2*^) were utilised to identify the variance in PA (mins/week) and SB (mins/week) that could be explained by the proposed model.

## Results

Participant information is presented in **Table 1**. Most participants were Caucasian (82.0%), women (72.7%), free of long-term mental health conditions (69.8%), and non-obese (65.9%).

**Table 1:** Participant characteristics. Data is presented as N (%) or mean ± SD.

**Age (y)**	**18** 1 (0.4)	**19** 40 (15.7)	**20** 72 (28.2)	**21** 65 (25.5)	**22–25** 51 (20.0)	**26–35** 20 (7.8)	**35+** 6 (2.4)
**Gender**	**F** 193 (75.7)	**M** 59 (23.1)	**Neither/ other/ prefer not to say** 3 (1.2)				
**Ethnicity**	**Black** 9 (3.5)	**Asian** 23 (9.0)	**Mixed** 9 (3.5)	**White** 209 (82.0)	**Other/ prefer not to say** 5 (2.0)		
**Anthropometry**	**Height (m)**	**Weight (kg)**	**BMI (kg/m** ^**2**^ **)**				
	1.68 ± 0.10	70.2 ± 18.1	24.9 ± 6.0				
**University year group**	**Y1** 3 (1.2)	**Y2** 88 (34.5)	**Y3** 108 (42.3)	**Y4** 36 (14.1)	**Other** 20 (7.8)		
**Diagnosed long-term mental health condition**	**Yes** 77 (30.2)	**No** 178 (69.8)					

BMI = body mass index; SD = standard deviation.

**Fig 2:**
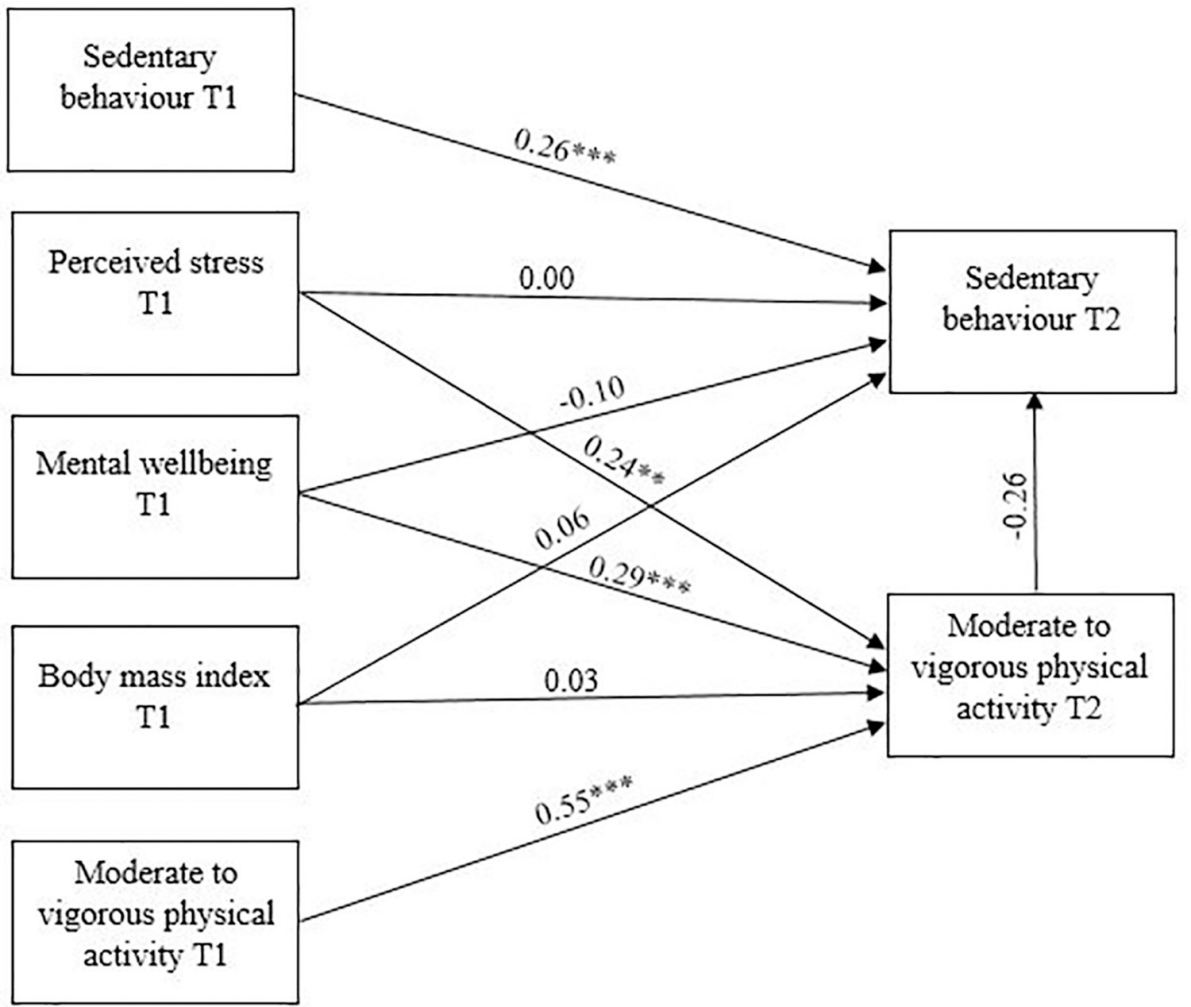
A summary of interactions between MWB, PS, BMI, SB, and MVPA pre-covid (T1), and SB and MVPA during covid (T2). The lines with arrowheads illustrate the direction of the affect. *** *P* < 0.001; ** *P* < 0.01.

**Fig 3:**
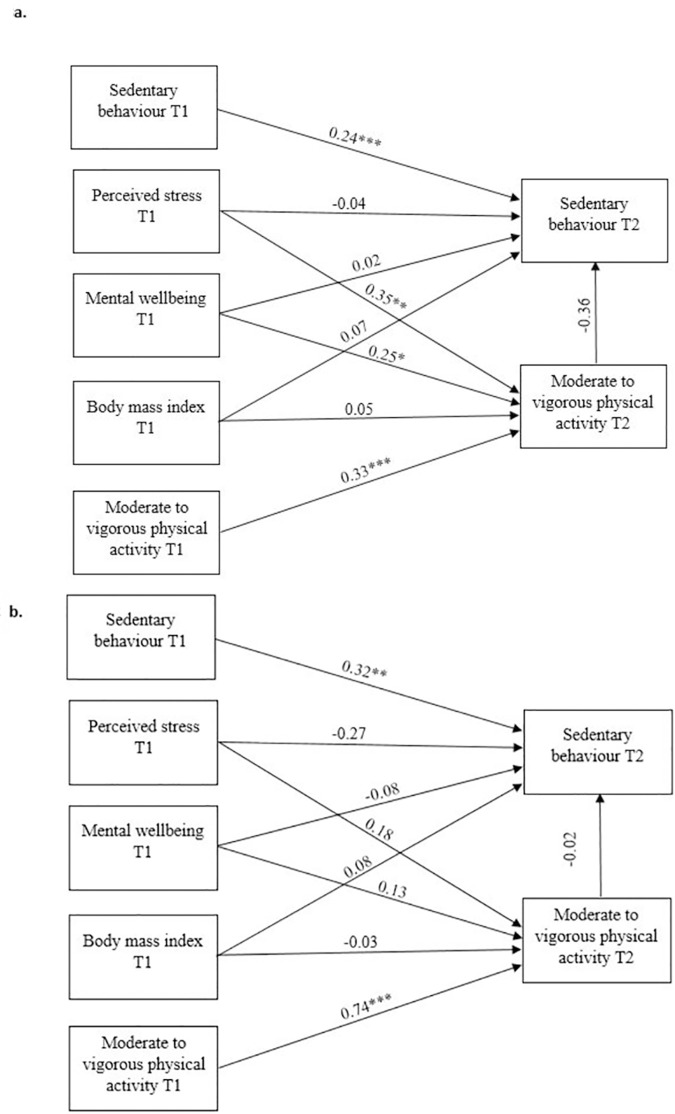
The final path analysis model for male **(a)** and female **(b)** university students. *** *P* < 0.001; ** *P* < 0.01; * *P* < 0.05.

The final model **(Fig 2)** details the interactions between SB, MWB, PS, BMI, and PA pre-covid, and SB and PA in UK university students nine months following the COVID-19 outbreak. The model fit values (*CMIN* = 0.10, *CMIN/DF* = 0.10, *CFI* = 1.00, *RMSEA* = 0.00) indicate the predictive ability of the model is acceptable and the model is a good fit. When separated by gender, the model was also a good fit for women and men (*CMIN* = 0.09, *CMIN/DF* = 0.05, *CFI* = 1.00, *RMSEA* = 0.00) **(Fig 3A & 3B)**.

Overall, pre-covid levels of MWB and PS did not directly affect SB during the pandemic (*β* = -0.10 and *P* = 0.18; *β* = 0.00 and *P* = 0.79) but pre-covid MWB and PS were positively related to PA during the pandemic (*β* = 0.29 and *P* < 0.01; *β* = 0.24 and *P* < 0.01). Examination of squared multiple correlations revealed that MWB prior to the pandemic explained 29% of the variance in PA during the pandemic, and pre-pandemic PS explained 23% of the variance in PA during the pandemic. Unsurprisingly, SB prior to the pandemic was positively related to SB during the pandemic (*β* = 0.26; *P* < 0.01), explaining 26% of the variance in SB during the pandemic. Also unsurprisingly, pre-pandemic PA was positively related to PA during the pandemic (*β* = 0.55; *P* < 0.01), explaining 55% of the variance in PA during the pandemic. However, BMI prior to the pandemic was not related to either SB or PA during the pandemic (*β* = 0.03 and *P* = 0.29; *β* = 0.06 and *P* = 0.56), and there was no mediating effect of any pre-pandemic variables through PA on SB during the pandemic (*β* = -0.26; *P* = 0.14) **(Fig 2)**.

Similar to the overall model, in men MWB and PS prior to the pandemic did not directly affect SB (*β* = -0.04 and *P* = 0.76; *β* = 0.02 and *P* = 0.86). However, men that had higher mental wellbeing and were more stressed prior to the pandemic engaged in greater amounts of PA during the pandemic (*β* = 0.35 and *P* < 0.01; *β* = 0.25 and *P* = 0.02). Additionally, in men, MWB prior to the pandemic explained 35% of the variance in PA during the pandemic, and pre-pandemic PS explained 25% of the variance in PA during the pandemic. Furthermore, SB prior to the pandemic was positively related to SB during the pandemic in men (*β* = 0.24; *P* < 0.01), explaining 24% of the variance in SB during the pandemic, and pre-pandemic PA was positively related to PA during the pandemic (*β* = 0.33; *P* < 0.01), explaining 33% of the variance in PA during the pandemic. However, BMI prior to the pandemic was not related to either SB or PA during the pandemic in men (*β* = 0.07 and *P* = 0.29; *β* = 0.07 and *P* = 0.44). Additionally, and in line with the overall model, there was no mediating effect of any pre-pandemic variables through PA on SB during the pandemic in men (*β* = -0.36; *P* = 0.10) **(Fig 3A)**.

In contrast to the overall model and the men model, in women no direct relationships were observed between MWB and PS pre-pandemic and PA (MWB: *β* = -0.18 and *P* = 0.20; PS: *β* = 0.13 and *P* = 0.93) or SB (MWB: *β* = -0.27 and *P* = 0.20; PS: *β* = -0.08 and *P* = 0.70) during the pandemic. Similarly, pre-pandemic BMI was not related to PA (*β* = -0.03 and *P* = 0.74) or SB (*β* = 0.08 and *P* = 0.52) during the pandemic. However, and as expected, SB prior to the pandemic was positively related to SB during the pandemic (*β* = 0.32 and *P* < 0.01), and PA prior to the pandemic was positively related to PA during the pandemic in women (*β* = 0.74 and *P* < 0.01). Squared multiple correlations indicated that SB pre-pandemic explained 32% of the variance and that PA prior to the pandemic explained 74% of the variance in PA during the pandemic in women. As per the main model and that for the men, there were no mediating effects of any pre-pandemic variables through PA on SB during the pandemic in women (*β* = -0.02 and *P* = 0.92) **(Fig 3B)**.

## Discussion

The current study used path analysis to investigate factors prior to the pandemic that influenced PA and SB during the pandemic. The findings of this longitudinal study demonstrate that having better MWB but higher PS prior to the pandemic was related to undertaking greater amounts of PA during the pandemic. However, when separated by gender, this relationship was only present in men and not in women providing further evidence that gender must be considered when developing effective intervention strategies to improve movement behaviours in university students [47].

Previously, several longitudinal studies had demonstrated that mental health status can impact movement behaviours in university students and young people [17, 19, 20, 23–25]. However, the findings of the current study provide novel insight and demonstrate that these relationships remain for PA despite the COVID-19 pandemic. Previously, it has been shown that having better psychological wellbeing can have a positive influence on PA and it appears that this relationship has not been altered within the context of the pandemic [19, 20]. Additionally, there is evidence to suggest that university students often utilise PA as a method of relieving stress during demanding periods [48]. As such, the notion that higher amounts of PS pre-pandemic were related to greater levels of PA during the pandemic may be expected given that students have significant changes to daily living which has led to dramatic increases in stress [27–33, 49].

However, in contrast, the findings of the current study suggest that the pandemic mitigated any previous relationships observed between mental health and SB in university students [17, 23, 25]. This could be largely due to the nature of the restrictions implemented during ‘lockdown’ periods. In the UK, despite the closure of indoor exercise settings (i.e., gyms, sports clubs) people were still able to conduct structured bouts of physical activity outside. As such, it is possible that psychological factors influencing PA prior to the pandemic also influenced PA during the pandemic, but not SB. Furthermore, movement restrictions imposed during the pandemic enforced prolonged periods of SB due to the lack of necessity to walk to classes and between campus buildings [32, 33, 50]. Therefore, it is possible that university students’ SB was increased irrespective of mental health status prior to the pandemic.

Similar reasoning may be applied to the lack of relationship between BMI prior to the pandemic and movement behaviours during the pandemic. Previously, it has been shown that body composition is longitudinally related to low PA [51] and high SB [7]. Additionally, higher BMI was associated with reduced PA during the pandemic in UK adults [52]. However, the imposition of COVID-related movement restrictions led to the impairment of movement behaviours irrespective of BMI status [53]. As such, it is likely that pre-COVID BMI did not have a profound influence on movement behaviours during the pandemic as supported by the findings of the current study. Instead, behavioural factors prior to the pandemic may have had a distinct influence on PA and SB in the context of COVID-19. Recent literature has suggested that pre-COVID behaviours or habits were directly related to PA levels during a period of government enforced restrictions [54]. This is further evidenced by the findings of the current study, illustrating that SB and PA prior to the pandemic had a direct positive influence on SB and PA during the pandemic respectively. Whilst perhaps unsurprising, this provides novel insight into behavioural determinants of movement behaviours in university students. Furthermore, no mediating influence of PA on SB during the pandemic was observed for any pre-pandemic variable. It is therefore likely that SB during the pandemic was largely influenced by COVID-specific factors largely generated from government-imposed movement restrictions [47].

Interestingly, when separated by gender, the positive relationships between pre-COVID MWB and PS, and MVPA during the pandemic were present in men but not in women. This further demonstrates the disparity between genders when assessing determinants of physical activity in young people [47]. Previously, it has been shown that male students tend to be more physically active than female students [18]. Furthermore, male students are stated to be more intrinsically motivated to exercise (i.e., for enjoyment, challenge, and competition) whereas female students depend on external factors (i.e., ill-health avoidance, weight management and appearance) [55–57]. It is therefore unsurprising that the COVID-19 pandemic appears to have negatively impacted PA to a greater extent in men compared to women [32]. The current study builds on this further by demonstrating that pre-COVID mental health status influenced PA in men but not in women during the pandemic. As such, future policy and interventions should consider previous mental health status when developing interventions aimed at improving PA in male students post-COVID [47]. However, further research is required to establish determinants of movement behaviours in female students.

A major strength of the present study is the prospective longitudinal study design. Inclusion of survey data prior to and during COVID-related restrictions (October 2019 & October 2020) has allowed the identification of pre-COVID factors that influenced PA and SB during COVID-related restrictions. This adds to existing literature by identifying psychological factors related to mental health that may have played a role in determining PA levels during the pandemic [32, 33]. This may be particularly important for university students given that impaired movement behaviours have been related to poorer academic performance [58]. To address this previous research has suggested that online videos based on self-determination theory could have a beneficial influence on movement behaviours in young people during the pandemic [59]. However, it is currently unknown whether these results would be reproducible in free living university students. Additionally, a text message-based intervention has been implemented in university students and whilst time spent sedentary did not change, participants responded positively to the intervention [60]. Taken together, these intervention strategies may provide an appropriate method of improving students’ movement behaviours, particularly following the lifting of COVID related restrictions. Although the UK no longer has limits on movement or social contact, the pandemic is expected to have a lasting influence on social, economic and cultural aspects of higher education institutions for years to come [37]. As such, the findings of the current study should be considered by researchers and universities when developing and implementing intervention strategies aimed at improving students’ movement behaviours.

The self-reported nature of surveys may lead to inaccuracies within the data, in particular an underestimation of time spent sedentary [61]. Additionally, the study did not assess all aspects of mental health that have previously been shown to be related to movement behaviours in university students such as anxiety, depression, and self-efficacy [62, 63]. As such, the present study is unable to fully explain the determinants of SB and PA in this population. However, these behaviours are likely defined by complex multifactorial systems [16, 17] which are extremely difficult to capture in a single study. Furthermore, attrition rate was high between T1 and T2, and women contributed a much greater response rate than men, highlighting the potential for self-selection bias and providing some explanation for the differences observed in the gender-based models. Furthermore, 255 students completed the survey at T2 from an initial sample of 946 at T1. It is therefore likely that a proportion of the respondents at T1 would have graduated the University by T2 and therefore been ineligible to respond. Overall, a high attrition rate is well-known in survey-based research investigating young adults and may therefore be unavoidable in studies of this nature [64–67].

## Conclusion

The current study used path analysis derived from SEM to identify longitudinal determinants of PA and SB in university students during the pandemic. The findings indicate that pre-COVID levels of mental wellbeing and perceived stress positively directly influenced levels of MVPA during the pandemic in university students. The longitudinal design and the statistical methods employed have enabled the current study to enhance current knowledge surrounding the importance of student mental wellbeing and stress management, particularly for future health related behaviours.

## References

[1] Allender S., Foster C., Scarborough P., & Rayner M. (2007). The burden of physical activity-related ill health in the UK. Journal of Epidemiology & Community Health, 61(4), 344–348. 10.1093/pubmed/fdr033 17372296 PMC2652953

[2] Heron L., O’Neill C., McAneney H., Kee F., Tully M.A., 2019. Direct healthcare costs of sedentary behaviour in the UK. J Epidemiol Community Health 73, 625–629. doi: 10.1136/jech-2018-211758 30910857

[3] Daw J., Margolis R., Wright L., 2017. Emerging Adulthood, Emergent Health Lifestyles: Sociodemographic Determinants of Trajectories of Smoking, Binge Drinking, Obesity, and Sedentary Behavior. J Health Soc Behav 58, 181–197. doi: 10.1177/0022146517702421 28661779 PMC5894852

[4] Castro O., Bennie J., Vergeer I., Bosselut G., Biddle S.J.H., 2020. How Sedentary Are University Students? A Systematic Review and Meta-Analysis. Prev Sci 21, 332–343. 10.1007/s11121-020-01093-8 31975312

[5] Rouse P.C., Biddle S.J.H., 2010. An ecological momentary assessment of the physical activity and sedentary behaviour patterns of university students. Health Education Journal 69, 116–125. 10.1177/0017896910363145

[6] Levitsky D. A., Halbmaier C. A., & Mrdjenovic G. (2004). The freshman weight gain: a model for the study of the epidemic of obesity. International journal of obesity, 28(11), 1435–1442. doi: 10.1038/sj.ijo.0802776 15365585

[7] Vainshelboim B., Brennan G. M., LoRusso S., Fitzgerald P., & Wisniewski K. S. (2019). Sedentary behavior and physiological health determinants in male and female college students. Physiology & behavior, 204, 277–282. doi: 10.1016/j.physbeh.2019.02.041 30831185

[8] Patel A. V., Bernstein L., Deka A., Feigelson H. S., Campbell P. T., Gapstur S. M., et al. (2010). Leisure time spent sitting in relation to total mortality in a prospective cohort of US adults. American journal of epidemiology, 172(4), 419–429. doi: 10.1093/aje/kwq155 20650954 PMC3590043

[9] Peltzer K., & Pengpid S. (2016). Leisure time physical inactivity and sedentary behaviour and lifestyle correlates among students aged 13–15 in the association of Southeast Asian nations (ASEAN) member states, 2007–2013. International journal of environmental research and public health, 13(2), 217. doi: 10.3390/ijerph13020217 26891312 PMC4772237

[10] King A. C., Parkinson K. N., Adamson A. J., Murray L., Besson H., Reilly J. J., et al. (2011). Correlates of objectively measured physical activity and sedentary behaviour in English children. The European Journal of Public Health, 21(4), 424–431. doi: 10.1093/eurpub/ckq104 20650946

[11] Brodersen N. H., Steptoe A., Williamson S., & Wardle J. (2005). Sociodemographic, developmental, environmental, and psychological correlates of physical activity and sedentary behavior at age 11 to 12. Annals of Behavioral Medicine, 29(1), 2–11. doi: 10.1207/s15324796abm2901_2 15677295

[12] Kypri K. Y. P., Cronin M., & Wright C. S. (2005). Do university students drink more hazardously than their non-student peers? Addiction, 100(5), 713–714. doi: 10.1111/j.1360-0443.2005.01116.x 15847629

[13] Harding J. (2011). Financial circumstances, financial difficulties and academic achievement among first-year undergraduates. Journal of Further and Higher Education, 35(4), 483–499.

[14] Bennett T. H., & Holloway K. R. (2015). Drug use among college and university students: Findings from a national survey. Journal of Substance Use, 20(1), 50–55.

[15] Holliman A. J., Waldeck D., Jay B., Murphy S., Atkinson E., Collie R. J., et al. (2021). Adaptability and social support: Examining links with psychological wellbeing among UK students and non-students. Frontiers in Psychology, 205. doi: 10.3389/fpsyg.2021.636520 33613406 PMC7894575

[16] Deliens T., Deforche B., De Bourdeaudhuij I., Clarys P., 2015. Determinants of physical activity and sedentary behaviour in university students: a qualitative study using focus group discussions. BMC Public Health 15, 201. doi: 10.1186/s12889-015-1553-4 25881120 PMC4349731

[17] Castro O., Bennie J., Vergeer I., Bosselut G., Biddle S.J.H., 2018. Correlates of sedentary behaviour in university students: A systematic review. Preventive Medicine 116, 194–202. doi: 10.1016/j.ypmed.2018.09.016 30266213

[18] Lerner J., Burns C., & De Róiste Á. (2011). Correlates of physical activity among college students. Recreational Sports Journal, 35(2), 95–106.

[19] Bai Y., Copeland W. E., Burns R., Nardone H., Devadanam V., Rettew J., et al. (2022). Ecological momentary assessment of physical activity and wellness behaviors in college students throughout a school year: Longitudinal naturalistic study. JMIR Public Health and Surveillance, 8(1), e25375. doi: 10.2196/25375 34982721 PMC8767478

[20] Doré I., O’loughlin J. L., Schnitzer M. E., Datta G. D., & Fournier L. (2018). The longitudinal association between the context of physical activity and mental health in early adulthood. Mental Health and Physical Activity, 14, 121–130. 10.1016/j.mhpa.2018.04.001

[21] Peterson N. E., Sirard J. R., Kulbok P. A., DeBoer M. D., & Erickson J. M. (2018). Sedentary behavior and physical activity of young adult university students. Research in nursing & health, 41(1), 30–38. doi: 10.1002/nur.21845 29315656 PMC10926845

[22] Zaccagni L., Barbieri D., & Gualdi-Russo E. (2014). Body composition and physical activity in Italian university students. Journal of Translational Medicine, 12, 1–9. 10.1186/1479-5876-12-120 24885945 PMC4025557

[23] Hoare E., Milton K., Foster C., Allender S., 2016. The associations between sedentary behaviour and mental health among adolescents: a systematic review. Int J Behav Nutr Phys Act 13, 108. doi: 10.1186/s12966-016-0432-4 27717387 PMC5055671

[24] Hamer M., Yates T., Sherar L.B., Clemes S.A., Shankar A., 2016. Association of after school sedentary behaviour in adolescence with mental wellbeing in adulthood. Preventive Medicine 87, 6–10. doi: 10.1016/j.ypmed.2016.02.021 26876629

[25] Babic M.J., Smith J.J., Morgan P.J., Eather N., Plotnikoff R.C., Lubans D.R., 2017. Longitudinal associations between changes in screen-time and mental health outcomes in adolescents. Mental Health and Physical Activity 12, 124–131. 10.1016/j.mhpa.2017.04.001

[26] Thorley C., 2017. Not by degrees: Improving student mental health in the UK’s universities. IPPR: London, UK.

[27] Cao W., Fang Z., Hou G., Han M., Xu X., Dong J., et al. 2020. The psychological impact of the COVID-19 epidemic on college students in China. Psychiatry Research 287, 112934. doi: 10.1016/j.psychres.2020.112934 32229390 PMC7102633

[28] Sahu P., 2020. Closure of Universities Due to Coronavirus Disease 2019 (COVID-19): Impact on Education and Mental Health of Students and Academic Staff. Cureus. doi: 10.7759/cureus.7541 32377489 PMC7198094

[29] Zhai Y., Du X., 2020. Addressing collegiate mental health amid COVID-19 pandemic. Psychiatry Research 288, 113003. doi: 10.1016/j.psychres.2020.113003 32315885 PMC7162776

[30] Bao Y., Sun Y., Meng S., Shi J., Lu L., 2020. 2019-nCoV epidemic: address mental health care to empower society. The Lancet 395, e37–e38. doi: 10.1016/S0140-6736(20)30309-3 32043982 PMC7133594

[31] Elmer T., Mepham K., Stadtfeld C., 2020. Students under lockdown: Comparisons of students’ social networks and mental health before and during the COVID-19 crisis in Switzerland. PLoS ONE 15, e0236337. doi: 10.1371/journal.pone.0236337 32702065 PMC7377438

[32] Savage M.J., James R., Magistro D., Donaldson J., Healy L.C., Nevill M., et al. 2020. Mental health and movement behaviour during the COVID-19 pandemic in UK university students: Prospective cohort study. Mental Health and Physical Activity 19, 100357. 10.1016/j.mhpa.2020.100357

[33] Savage M.J., Hennis P.J., Magistro D., Donaldson J., Healy L.C., James R.M., 2021. Nine Months into the COVID-19 Pandemic: A Longitudinal Study Showing Mental Health and Movement Behaviours Are Impaired in UK Students. IJERPH 18, 2930. doi: 10.3390/ijerph18062930 33809313 PMC7999965

[34] Li Y., Wang A., Wu Y., Han N., & Huang H. (2021). Impact of the COVID-19 pandemic on the mental health of college students: a systematic review and meta-analysis. Frontiers in psychology, 12, 669119. doi: 10.3389/fpsyg.2021.669119 34335381 PMC8316976

[35] Khan M. A., Menon P., Govender R., Samra A. M. A., Allaham K. K., Nauman J., et al. (2022). Systematic review of the effects of pandemic confinements on body weight and their determinants. British Journal of Nutrition, 127(2), 298–317. doi: 10.1017/S0007114521000921 33706844 PMC8376925

[36] Salway R, Foster C, de Vocht F, Tibbitts B, Emm-Collison L, House D, et al. Accelerometer-measured physical activity and sedentary time among children and their parents in the UK before and after COVID-19 lockdowns: a natural experiment. International Journal of Behavioral Nutrition and Physical Activity. 1, 1–4. 10.1186/s12966-022-01290-4 PMC9107948 35570265

[37] The British Academy, 2021. The COVID decade: Understanding the long-term societal impacts of COVID-19. London, UK: The British Academy.

[38] Von Elm E, Altman DG, Egger M, Pocock SJ, Gøtzsche PC, Vandenbroucke JP. (2007). The Strengthening the Reporting of Observational Studies in Epidemiology (STROBE) statement: guidelines for reporting observational studies. The Lancet. 370(9596):1453–7. 10.1016/S0140-6736(07)61602-X 18064739

[39] Coleman K. J., Ngor E., Reynolds K., Quinn V. P., Koebnick C., Young D. R., et al. (2012). Initial validation of an exercise “vital sign” in electronic medical records. Medicine & Science in Sports & Exercise, 44(11), 2071–2076. 22688832 10.1249/MSS.0b013e3182630ec1

[40] Armstrong T., Bull F., 2006. Development of the World Health Organization Global Physical Activity Questionnaire (GPAQ). J Public Health 14, 66–70. 10.1007/s10389-006-0024-x

[41] Cohen S., Kamarck T., Mermelstein R., 1983. A Global Measure of Perceived Stress. Journal of Health and Social Behavior 24, 385. 10.2307/2136404 6668417

[42] Tennant R., Hiller L., Fishwick R., Platt S., Joseph S., Weich S., et al. 2007. The Warwick-Edinburgh Mental Well-being Scale (WEMWBS): development and UK validation. Health Qual Life Outcomes 5, 63. doi: 10.1186/1477-7525-5-63 18042300 PMC2222612

[43] Denovan A., Dagnall N., Dhingra K., Grogan S. (2019). Evaluating the Perceived Stress Scale among UK university students: implications for stress measurement and management. Studies in Higher Education 44, 120–133. 10.1080/03075079.2017.1340445

[44] Hair J, Alamer A. Partial Least Squares Structural Equation Modeling (PLS-SEM) in second language and education research: Guidelines using an applied example. (2022). Research Methods in Applied Linguistics., 1;1(3):100027.

[45] Savalei V., & Kolenikov S. (2008). Constrained versus unconstrained estimation in structural equation modeling. Psychological methods, 13(2), 150. doi: 10.1037/1082-989X.13.2.150 18557683

[46] Kline RB. Principles and practice of structural equation modeling. The Guilford Press; 1998.

[47] Biddle S. J., Atkin A. J., Cavill N., & Foster C. (2011). Correlates of physical activity in youth: a review of quantitative systematic reviews. International review of sport and exercise psychology, 4(1), 25–49. 10.1080/1750984X.2010.548528

[48] Bland H. W., Melton B. F., Bigham L. E., & Welle P. D. (2014). Quantifying the impact of physical activity on stress tolerance in college students. College student journal, 48(4), 559–568.

[49] Aristovnik A., Keržič D., Ravšelj D., Tomaževič N., Umek L., 2020. Impacts of the COVID-19 Pandemic on Life of Higher Education Students: A Global Perspective. Sustainability 12, 8438. 10.3390/su12208438 PMC8634691 34869802

[50] Barkley J. E., Lepp A., Glickman E., Farnell G., Beiting J., Wiet R., et al. (2020). The acute effects of the COVID-19 pandemic on physical activity and sedentary behavior in university students and employees. International journal of exercise science, 13(5), 1326. 33042377 10.70252/QCVG2516 PMC7523895

[51] Fan J. X., Brown B. B., Hanson H., Kowaleski-Jones L., Smith K. R., & Zick C. D. (2013). Moderate to vigorous physical activity and weight outcomes: does every minute count? American Journal of Health Promotion, 28(1), 41–49. doi: 10.4278/ajhp.120606-QUAL-286 23458375 PMC5512715

[52] Robinson E., Boyland E., Chisholm A., Harrold J., Maloney N. G., Marty L., et al. (2021). Obesity, eating behavior and physical activity during COVID-19 lockdown: A study of UK adults. Appetite, 156, 104853. doi: 10.1016/j.appet.2020.104853 33038479 PMC7540284

[53] Alves J. M., Yunker A. G., DeFendis A., Xiang A. H., & Page K. A. (2021). BMI status and associations between affect, physical activity and anxiety among US children during COVID‐19. Pediatric Obesity, 16(9), e12786. 10.1111/ijpo.12786 33720550 PMC8250275

[54] Knight R.L., McNarry M.A., Sheeran L., Runacres A.W., Thatcher R., Shelley J., et al. 2021. Moving Forward: Understanding Correlates of Physical Activity and Sedentary Behaviour during COVID-19—An Integrative Review and Socioecological Approach. IJERPH 18, 10910. doi: 10.3390/ijerph182010910 34682653 PMC8535281

[55] Lauderdale M.E., Yli-Piipari S., Irwin C.C., Layne T.E., 2015. Gender Differences Regarding Motivation for Physical Activity Among College Students: A Self-Determination Approach. TPE. 10.18666/TPE-2015-V72-I5-4682

[56] Egli T., Bland H.W., Melton B.F., Czech D.R., 2011. Influence of Age, Sex, and Race on College Students’ Exercise Motivation of Physical Activity. Journal of American College Health 59, 399–406. doi: 10.1080/07448481.2010.513074 21500059

[57] Gao Z., Xiang P., 2008. College Students’ Motivation Toward Weight Training: An Application of Expectancy-Value Model. Journal of Teaching in Physical Education 27, 399–415. 10.1123/jtpe.27.3.399

[58] Felez-Nobrega M., Hillman C.H., Dowd K.P., Cirera E., Puig-Ribera A., 2018. ActivPAL^TM^ determined sedentary behaviour, physical activity and academic achievement in college students. Journal of Sports Sciences 36, 2311–2316. 10.1080/02640414.2018.1451212 29533713

[59] McDonough D. J., Helgeson M. A., Liu W., & Gao Z. (2022). Effects of a remote, YouTube-delivered exercise intervention on young adults’ physical activity, sedentary behavior, and sleep during the COVID-19 pandemic: Randomized controlled trial. Journal of Sport and Health Science, 11(2), 145–156. doi: 10.1016/j.jshs.2021.07.009 34314877 PMC8487769

[60] Keahey R., White N., Duchesne A., Pelletier C.A., 2021. A theory-grounded text message–based intervention to reduce sedentary behaviour in university students. Health Education Journal 80, 672–685. 10.1177/00178969211007163

[61] Prince S.A., Cardilli L., Reed J.L., Saunders T.J., Kite C., Douillette K., et al. 2020. A comparison of self-reported and device measured sedentary behaviour in adults: a systematic review and meta-analysis. Int J Behav Nutr Phys Act 17, 31. doi: 10.1186/s12966-020-00938-3 32131845 PMC7055033

[62] Lee E., Kim Y., 2019. Effect of university students’ sedentary behavior on stress, anxiety, and depression. Perspect Psychiatr Care 55, 164–169. doi: 10.1111/ppc.12296 29797324 PMC7818186

[63] Rollo S., Gaston A., & Prapavessis H. (2016). Cognitive and motivational factors associated with sedentary behavior: A systematic review. AIMS Public Health, 3(4), 956. 10.3934/publichealth.2016.4.956. doi: 10.3934/publichealth.2016.4.956 29546206 PMC5690416

[64] Porter S.R., Whitcomb M.E., 2005. Non-response in student surveys: The Role of Demographics, Engagement and Personality. Res High Educ 46, 127–152. 10.1007/s11162-004-1597-2

[65] Eysenbach G., 2005. The Law of Attrition. J Med Internet Res 7, e11. doi: 10.2196/jmir.7.1.e11 15829473 PMC1550631

[66] Kelders S.M., Kok R.N., Ossebaard H.C., Van Gemert-Pijnen J.E., 2012. Persuasive System Design Does Matter: a Systematic Review of Adherence to Web-based Interventions. J Med Internet Res 14, e152. doi: 10.2196/jmir.2104 23151820 PMC3510730

[67] Rübsamen N., Akmatov M.K., Castell S., Karch A., Mikolajczyk R.T., 2017. Factors associated with attrition in a longitudinal online study: results from the HaBIDS panel. BMC Med Res Methodol 17, 132. doi: 10.1186/s12874-017-0408-3 28859617 PMC5580321

